# Clinical Prediction Score for Bowel Resection in Pediatric Intussusception in Resource-Limited Settings

**DOI:** 10.7759/cureus.117777

**Published:** 2026-10-11

**Authors:** Akinlabi E Ajao, Felix O Kumolalo, Dare I Olulana, Taiwo A Lawal, Olakayode O Ogundoyin

**Affiliations:** 1 Pediatric Surgery, Bowen University Teaching Hospital, Ogbomosho, GBR; 2 Pediatric Surgery, University College Hospital, Ibadan, NGA; 3 Pediatric Surgery, University of Medical Sciences, Ondo, NGA

**Keywords:** bowel resection, clinical score, diagnosis, intussusception, low- and middle-income countries

## Abstract

Background and aim

Delayed diagnosis and referral are major contributing factors to morbidity and mortality in childhood intussusception in low- and middle-income countries. This study aimed to develop a score for predicting the need for bowel resection in childhood intussusception in low-resource settings.

Methods

This retrospective study was conducted at the pediatric surgical units of three institutions in South-West Nigeria. This study included children aged between 0 and 36 months managed for intussusception between January 2006 and December 2019. Statistical analysis was done using the chi-square, Fisher’s exact test, Mann-Whitney U test, and multivariate logistic regression depending on data type and distribution.

Results

A total of 212 children were included in this study, and the need for bowel resection was assessed. Following univariate analysis, statistically significant variables were included in multivariate analysis. Five variables, abdominal distension, fever, age, abdominal pain, and anemia independently predicted the outcome of bowel resection. The regression analysis was used to compute the scoring system, giving 13 points for abdominal distension, 10 for fever, 9 for abdominal pain, 10 for anemia, and -1 for each month of age. There was a positive correlation between the scores and the risk of bowel resection (p<0.001). The area under the receiver operating characteristic (ROC) curve for this score, describing its prediction ability, was 74.8% (p<0.0001).

Conclusion

The scoring system predicts the need for bowel resection in children with intussusception. This can promote early referral of pediatric intussusception to the pediatric surgeon, who in turn can be guided to operate early in patients who may require bowel resection.

## Introduction

Intussusception can easily be diagnosed in children using ultrasonography following clinical suspicion [1]. However, in low- and middle-income countries (LMICs), late presentation and referral delays limit access to treatment [2-4]. These limit treatment options to surgery, as non-operative reduction (NOR) may become ineffective or hazardous [5-7].

For the surgeon, making a decision on whether to attempt NOR or to proceed directly to operative management may be a difficult one. Bearing in mind that early intervention may significantly impact outcome, an early decision becomes important in patients presenting late in resource-limited settings. In developing countries, where there are limited trained professionals and restricted material resources, there is further delay in the management of surgical conditions due to logistical problems. In this environment, setting up for non-operative management of intussusception presents significant challenges and delays. This logistical delay could further delay management in patients who would eventually require surgical treatment. Therefore, there is a need for a reliable means to predict the need for bowel resection, which could circumvent delays caused by unnecessary attempts at reduction in this environment or at least guide decision-making.

The limitation of imaging to predict non-operative reducibility of intussusception makes it imperative that an alternative predictive measure be considered in these cases. Attempts have been made to predict failure of NOR, bowel compromise, and need for bowel resection in intussusception using clinical, radiological, and serum markers [8-12].

The present study aimed to develop a scoring system to predict the likelihood of requiring bowel resection in children with intussusception using clinical parameters. The authors believe this would help guide decisions on whether to consider an attempt at NOR, especially in delayed presentation.

## Materials and methods

This was a retrospective review of patients managed for intussusception between January 2006 and December 2019 at three tertiary care hospitals in the South-West region of Nigeria. This study was part of a broader study on childhood intussusception conducted in all three institutions with institutional review board approval number NHREC/12/04/2012 from the Bowen University Research Ethics Committee. The study included consecutive children aged 36 months and below, diagnosed with intussusception during the study period. We excluded patients who had obvious features of peritonitis at presentation, which meant they did not have the option of non-surgical reduction, were taken directly to theatre for surgery, and were also more likely, theoretically, to require a bowel resection. We also excluded those who were taken away from the hospital by their caregivers against medical advice, in whom the outcome of interest could not be determined thereafter.

Data were collected from clinical records as recorded by the managing teams in the patients’ paper medical records. The main explanatory variables were age, sex, duration of symptoms from the first symptom, and the presenting clinical symptoms, including abdominal pain, vomiting, bloody stool, diarrhea, and preceding history of upper respiratory tract infection. Clinical signs elicited by the attending surgical staff were also recorded, and these included general physical examination findings, abdominal distension, presence of mass, rectal bleeding, anemia status, and recorded clinical observation parameters.

Definition of terms

Intussusception was defined as the radiologic and/or intraoperative finding of bowel invagination into an adjacent segment. A senior registrar or consultant radiologist made the radiological diagnosis using ultrasound scans. Surgical procedures were performed by either a senior registrar or a consultant in pediatric surgery. We collected records of patients with this diagnosis from admission records on the general pediatrics and pediatric surgical wards and from surgical theater records.

Abdominal distension was considered present when the clinical examination documented a visibly or palpably distended abdomen or a significantly full abdomen at presentation, as determined by the clinician. Anemia was defined as a hematocrit of <30%. The outcome of interest was the need for bowel resection as part of definitive treatment. Information was also obtained on the indications for bowel resection in this cohort of patients.

Statistics

Univariate analyses of categorical factors related to bowel resection were done using chi-square and Fisher’s exact statistics. Continuous variables were assessed using the Mann-Whitney U test. Variables that were statistically significant in univariate analysis were included in a multivariate logistic regression analysis. Independent predictors of bowel resection were then included in the final model, which was used to develop the risk assessment score. We assessed the linearity assumption for age (in months) in the logit using the Box-Tidwell procedure. The Hosmer-Lemeshow test statistic was used to test the goodness of fit of the final model.

The regression coefficients of significant variables from the multivariate logistic regression analysis were converted to transformed coefficients by dividing the coefficients by the smallest coefficient in the model. These transformed coefficients were then rounded up to the nearest integer to give the weighted scores for the identified indicators. These weighted scores were added together to give a total score to predict the need for bowel resection in a child with intussusception. A receiver operating characteristic (ROC) curve was plotted using the obtained total scores to determine the predictive ability of the model to predict the eventual requirement for bowel resection based on the final treatment as identified in the data. The final scores were then categorized based on the calculated risk for bowel resection. Statistical analysis was performed using IBM SPSS version 23 (Armonk, NY: IBM Corp.) for Windows. The level of statistical significance was set at p<0.1.

## Results

During the study period, 229 patients who met the inclusion criteria were recruited from three institutions. However, only 212 were included in the final analysis after excluding those with incomplete data on the outcome of interest. The median age of the children was seven months, with a range of one to 36 months. The cohort included 131 (61.8%) males and 81 (38.2%) females. The median duration of symptoms was three days, with a range of 2 h to 28 days. A total of 113 (53.3%) patients required eventual bowel resection, while 99 (46.7%) did not require bowel resection for treatment. Table 1 summarizes the indications for bowel resection in this cohort. Univariate analysis identified nine factors significantly associated with bowel resection. These factors included age, duration of symptoms, fever, abdominal pain, abdominal distension, heart rate, rectal mass, anemia, and treatment for more than 24 h at a peripheral hospital (Table 2). The final assessment score included only the five factors found statistically significant following multivariate logistic regression analysis. These factors were abdominal distension, fever, age, abdominal pain, and anemia (Table 3). The Hosmer-Lemeshow test of goodness of fit for this model showed no evidence of poor fit (p=0.628).

**Table 1:** Indications for bowel resection among children undergoing treatment for intussusception.

Indications	Frequency	Percentage
Gangrene	89	78.8
Non-reducibility	14	12.4
Pathologic lead point	9	7.9
Intestinal perforation	1	0.9
Total	113	100.0

**Table 2:** Comparison of the clinical characteristics of intussusception cases based on their requirement of bowel resection. *Values are presented as median and interquartile range, and analysis was done using the Mann-Whitney U test. **Fever was defined as temperature ≥37.5°C. ***Anemia was defined as hematocrit <30%. ****P<0.1 considered significant. RTI: respiratory tract infection

Variables	Bowel resection		χ^2^	p-Value
	Yes, n (%)	No, n (%)		
Age (months)*	6 (3)	7 (5)	-	0.058***
Sex				
Male	64 (48.9)	67 (51.1)	2.72	0.10
Female	49 (60.5)	32 (39.5)	-	-
Duration of symptoms (days)*	4 (3)	2 (3)	-	0.023****
Abdominal pain	87 (57.6)	64 (42.4)	4.93	0.03****
Abdominal distension	76 (67.9)	36 (32.1)	22.36	<0.001****
Vomiting	107 (54.3)	90 (45.7)	3.06	0.08****
Bloody stool	107 (53.8)	92 (46.2)	0.28	0.59
Preceding diarrhea	9 (42.9)	12 (57.1)	1.29	0.26
Recent RTI	22 (57.9)	16 (42.1)	0.02	0.90
Heart rate (beats per min)*	148 (34)	129 (79)	-	0.006****
Treatment for >24 h at a peripheral hospital	51 (68.0)	24 (32.0)	8.76	0.003****
Abdominal mass	53 (54.6)	44 (45.4)	0.07	0.79
Rectal mass	31 (73.8)	11 (26.2)	7.99	0.005****
Rectal protrusion	11 (64.7)	6 (35.3)	0.76	0.38
Fever**	91 (59.5)	62 (40.5)	9.46	0.002****
Dehydration	69 (50.4)	68 (49.6)	1.02	0.31
Anemia***	50 (64.1)	28 (35.9)	6.04	0.014****

**Table 3:** Multivariate independent predictors of bowel resection in pediatric intussusception. *Age was treated as a continuous variable. **P<0.1 was considered significant. Variables included in the analysis were selected based on statistical significance at p<0.1. "Ref" denotes the reference category against which the other value is compared. "β" represents the regression coefficient.

Risk factors	β	OR	95% CI	p-Value
Abdominal distension				
Yes	0.988	2.685	1.386-5.199	0.003**
No (Ref)	-	-	-	-
Fever				
Yes	0.738	2.091	0.954-4.582	0.065**
No (Ref)	-	-	-	-
Age (months)*	-0.075	0.928	0.861-1.0	0.049**
Abdominal pain				
Yes	0.677	1.968	0.919-4.215	0.081**
No (Ref)	-	-	-	-
Anemia				
Yes	0.745	2.106	1.069-4.145	0.031**
No (Ref)	-	-	-	-

The final score was developed based on the following logistic regression model:

$\ln\left(\frac{p}{1-p}\right) = \alpha + \beta_1 X_1 + \beta_2 X_2 + \cdots + \beta_n X_n$

$p = \frac{1}{1 + e^{-(\alpha + \beta_1 X_1 + \beta_2 X_2 + \beta_3 X_3 + \beta_4 X_4 + \beta_5 X_5)}}$

Here, p is the probability of eventual bowel resection at treatment, β represents the regression coefficient of the variable, and Xi are the independent (or explanatory) variables. The regression coefficients of these factors were converted into weighted scores by dividing the coefficients by the smallest coefficient in the model, and these were subsequently rounded up to the nearest integers as shown in Table 4. The risk assessment score for each patient was computed using the formula below.

$\mathrm{Score} = 13 \times \text{abdominal distension} + 10 \times \mathrm{fever} + 10 \times \mathrm{anemia} + 9 \times \text{abdominal pain} - \text{age (months)}$

**Table 4:** Scoring system for the need for bowel resection in pediatric intussusception based on clinical parameters. *Age was treated as a continuous variable. **Transformed coefficients were obtained by dividing the regression coefficients (β) obtained in Table 3 by the smallest coefficient in the model (i.e., 0.075).

Predictors	Transformed coefficient**	Score
Abdominal distension		
Yes	13.17	13
No	-	0
Fever		
Yes	9.84	10
No	-	0
Age (months)*	-1.00	-1
Abdominal pain		
Yes	9.02	9
No	-	0
Anemia		
Yes	9.93	10
No	-	0
Maximum score	-	41
Least possible score	-	-36

Definitions

“Abdominal distension” was defined as 1 if the patient had abdominal distension and 0 if not. “Fever” was defined as 1 if the patient’s temperature was 37.5°C or higher and 0 if less than 37.5°C. “Abdominal pain” was defined as 1 if the patient had abdominal pain that may be evidenced by excessive irritability, episodic crying, and pulling up of legs, and 0 if this was absent. “Anemia” was defined as 1 if the hematocrit was less than 30% and 0 if the hematocrit was 30% or more. “Age” was defined as the child's age in months, up to a maximum of 36 months.

The minimum possible score from this formula is -36, and the maximum score is 41, as the maximum age of patients in the present study was restricted to 36 months. The differing risk of bowel resection as a function of the risk score is shown in Figure 1. As shown in the figure, patients with scores of -5 and less had a 7% risk of bowel resection; those with scores of -4 to 14 had a risk of 37%; those with scores of 15-26 had a risk of 57%; while those with scores of 27 and above had a risk of 80%. This model demonstrates good predictive ability, as shown by the area under the ROC curve (AUC), which was 74.8%, and this was statistically significant (95% CI: 0.677-0.819; p<0.0001) (Figure 2).

**Figure 1:**
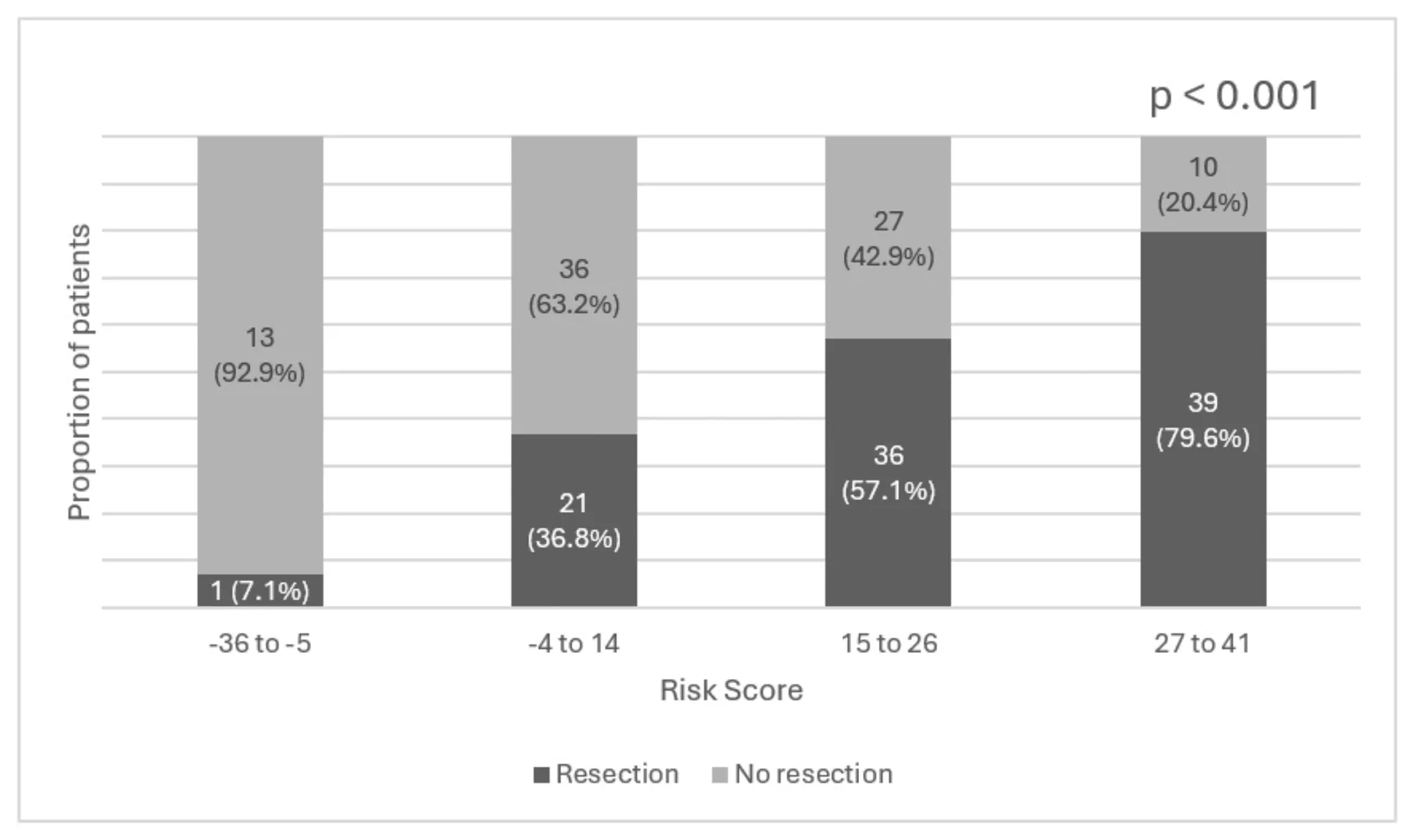
Chart showing the proportion of patients requiring bowel resection within the different risk assessment score categories.

**Figure 2:**
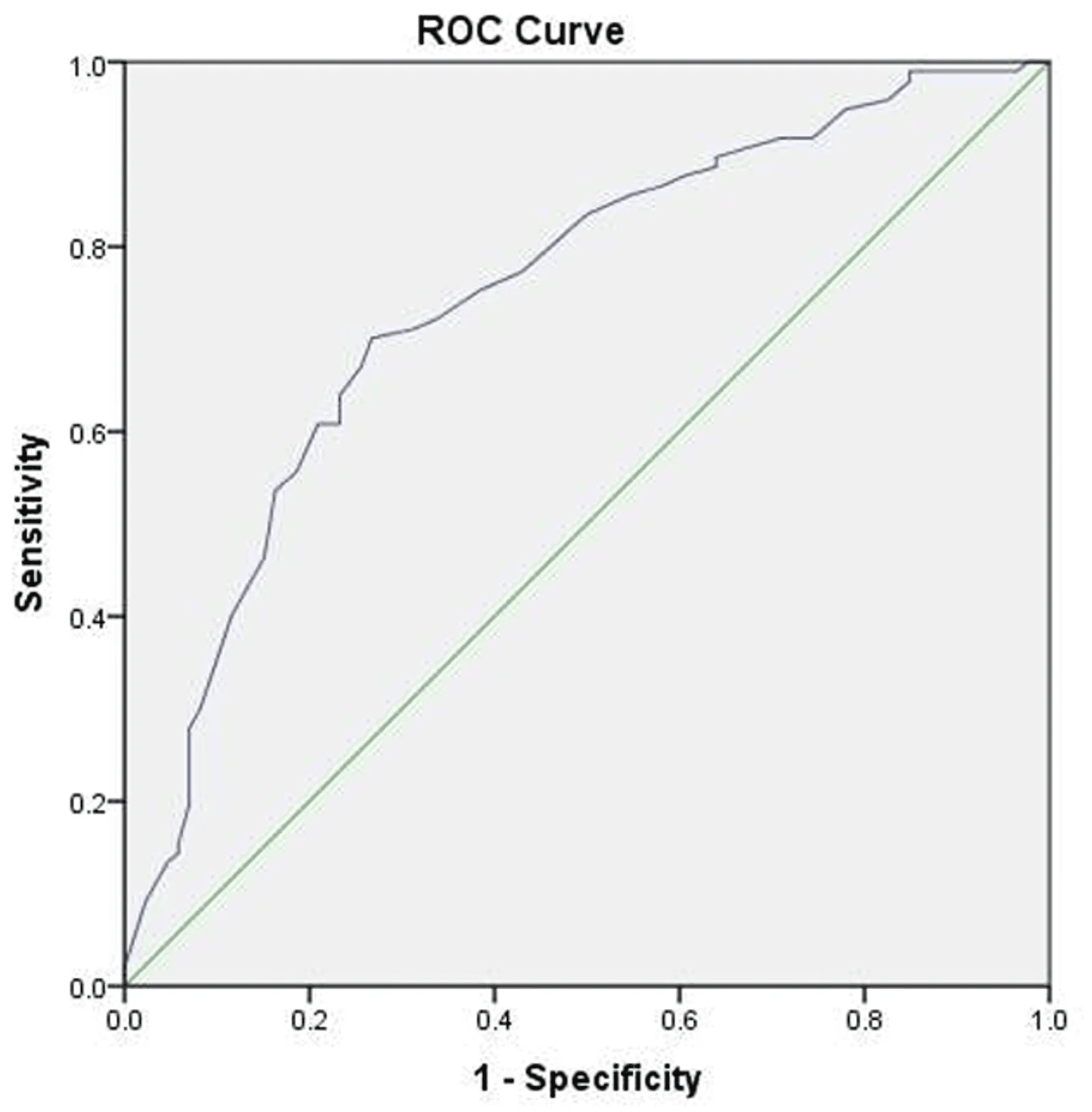
Receiver operating characteristic (ROC) curve for the need for bowel resection in children with intussusception predicted by the new scoring system. Area under the receiver operating characteristic curve (AUC) = 0.748; 95% confidence interval (CI) = 0.677-0.819; p<0.0001. “1 - specificity” represents the false-positive rate. The ROC curve plots sensitivity on the y-axis against 1 - specificity (false-positive rate) on the x-axis.

## Discussion

Early diagnosis and treatment are important in reducing the morbidity and mortality associated with childhood intussusception. Delayed presentation of patients in low- and middle-income countries (LMICs) has been variously alluded to and mostly ascribed to ignorance among parents or caregivers [2,3,12]. However, some delays are due to failure of healthcare givers to make a diagnosis or to make it early enough. In some cases, there is over-dependence on radiological investigations, which may be delayed either due to non-availability of radiologists or financial constraints.

Despite the usual delayed presentation at pediatric surgical units in LMICs, attempts are still being made to treat this condition using non-operative reduction (NOR). Although some studies have suggested a limit of 24-48 h, there has been no consensus on the duration of symptoms that absolutely contraindicates this mode of treatment [9,13]. NOR has been reported to be successful even in patients presenting up to five days after onset of symptoms [14]. There is, however, the need to reduce further delay in those patients who may ultimately require bowel resection as a result of intestinal gangrene, perforation, or non-reducibility of the intussusception. The ability to predict the need for bowel resection in these cases may be used to prevent any delay that may result from an unnecessary attempt at NOR.

This study developed a scoring system using clinical information and bedside investigation to predict the need for bowel resection in children with intussusception. The presence of four of the included parameters was associated with an increased risk of bowel resection. Patients with abdominal pain and distension, fever, and anemia had a significantly higher risk of bowel resection, which was then dependent on their age. Older age conferred some protection, and this was reported in an earlier series in Ibadan [11]. In that study, patients younger than 12 months were three times as likely as those older than 12 months to require bowel resection. It was hypothesized that this observation could have been due to increased susceptibility of infants to shock with its attendant compromise of the splanchnic circulation [11]. In the present study, each month of age reduced the risk of bowel resection by 7.2% (Table 3).

The five parameters of abdominal pain, anemia, abdominal distension, age, and fever (PADAF) can be obtained by primary care physicians, and the score can be computed quickly. The few parameters in this score and the fact that these parameters are the usual information obtained in this kind of condition would make the score system an easy-to-use tool for the first-contact physician. For the pediatric surgeon, it becomes a ready guide for decision-making when confronted with children with intussusception who may present beyond the traditional 48-72 h proposed by many as the threshold for abandoning NOR [13]. Another strength of this score is that the parameters are factors that can be objectively obtained and hence not immediately subject to observer bias.

From our literature search, there was a paucity of work on scoring systems to predict the need for resection in intussusception. There has, however, been some work done on predicting failure of NOR or the need for operative reduction, especially in developed countries [8,15]. An earlier study explored the use of the serum biochemical marker, intestinal fatty acid binding protein (I-FABP), as a preoperative predictor of intestinal necrosis in intussusception [12]. This biomarker could eliminate delays in treatment for patients who would require bowel resection because of bowel compromise. However, in addition to those requiring resection because of bowel gangrene, the new scoring system is expected to include other indications for bowel resection in intussusception such as non-reducibility, presence of pathologic lead points, and some cases of intestinal perforation. The new scoring system can therefore be used as a rapid initial test before obtaining such biochemical assays, especially in LMICs.

The scoring system was based on a regression model that showed good fit, as evidenced by the Hosmer-Lemeshow test of goodness of fit. It also showed good predictive ability with an area under the ROC curve (AUC) of 74.8% and displayed an identifiable trend between risk scores and the need for bowel resection. Patients with scores above 26 had an 80% risk of requiring bowel resection, while those with scores less than -4 had only a 7% risk.

Limitations

As a retrospective analysis, this study had the inherent problem of missing data, which may have introduced bias in data collection, computation, and analysis. The study also used the same cohort to validate the score and therefore requires external validation for generalizability.

## Conclusions

The risk of bowel resection in children with intussusception can be easily assessed using a scoring system that utilizes readily available patient information. It would be useful for early referral by first-contact physicians and as a guide to pediatric surgeons on the initial line of management. It would prevent delay from unnecessary attempts at NOR and facilitate early surgical intervention in high-risk patients, especially those presenting after 48 h of symptom onset. However, a prospective external validation of this score model will be required to improve generalizability.
